# Changes in physical activity and sleep habits among adults in Russian Federation during COVID-19: a cross-sectional study

**DOI:** 10.1186/s12889-021-10946-y

**Published:** 2021-05-11

**Authors:** Anna V. Kontsevaya, Dinara K. Mukaneeva, Azaliia O. Myrzamatova, Anthony D. Okely, Oxana M. Drapkina

**Affiliations:** 1 Department of Public Health Promotion, National Medical Research Centre for Therapy and Preventive Medicine of the Ministry of Health of Russia, Bld. 10, Petroverigskiy Lane, Moscow, 101990 Russia; 2 Early Start and Illawarra Health & Medical Research Institute, University of Wollongong, Wollongong, NSW Australia

**Keywords:** Movement behaviours, Pandemic, Environments, Adults, Russia

## Abstract

**Background:**

The aim of this study was to evaluate the impact of COVID-19 on the levels of physical activity (PA) and sleep and to examine specific COVID-19 factors that may be associated with changes in PA and sleep among adults in Russia.

**Methods:**

Cross-sectional data were collected during the period of tightest restrictions between 26 April 2020 and 6 June 2020. Eligible participants included all Russian adults aged 18 years and over. Participants reported their sleep patterns and problems, frequency and duration of walking, moderate- and vigorous-intensity PA, and muscle strengthening activities before COVID-19 and during the past 7 days. Access to an outdoor green space and fitness centres, use of online resources, adherence to self-isolation recommendations and other preventive measures from Ministry of Health were self-reported.

**Results:**

The sample included 2432 participants from 62 regions, 83% of who were female. There was a significant decline in the number of days per week participants reported not getting enough sleep (3.21 ± 2.44 to 2.86 ± 2.57; *P* < 0.001); participants also reported an increase in the number of days per week they had trouble falling asleep (1.70 ± 2.24 to 2.13 ± 2.48; *P* < 0.001). The proportion of participants who met the WHO Guidelines for PA declined from 68 to 49% (*P* < 0.001). The proportion who participated in muscle strengthening activities for 2 or more days per week declined from 53 to 45% (*P* < 0.001).

**Conclusion:**

Compared with before COVID-19, PA and sleep hygiene were adversely affected during COVID-19. Awareness of factors associated with these declines will assit policymakers in developing strategies to mitigate the negative lifestyle behaviours that have manifested during the COVID-19 confinement.

**Supplementary Information:**

The online version contains supplementary material available at 10.1186/s12889-021-10946-y.

## Key-points

1. Compared with before COVID-19, physical activity and sleep habits were adversely affected during COVID-19.
2. Awareness of factors associated with these declines will assit policymakers in developing strategies to mitigate the negative lifestyle behaviours that have manifested during the COVID-19 confinement.
3. Effective health promotion strategies directed at adopting or maintain positive health-related behaviours such as targeted social media messaging and balanced media reporting, should be used to reduce participant burden during these unprecedented times.

## Background

Physical activity (PA) is an important determinant of health [1], and is associated with all-cause mortality [2], risk of cardiovascular diseases and diabetes [3]. In November 2020 the World Health Organization (WHO) issued updated guidelines which recommend the amount of PA necessary to maintain health. These guidelines recommend that adults should be engaged in 150 min of moderate PA or 75 min of vigorous PA per week [4]. This recommendation is confirmed by systematic reviews and meta-analyses of studies which show that meeting the WHO guidelines resulted in significant reduction of cardiovascular events risk, cardiovascular mortality, and incidence of type 2 diabetes [5]. The proportion of adults in Russia who meet the WHO PA guidelines is only 30% [4], a figure not dissimilar with global rates [6].

The outbreak of novel coronavirus (COVID-19) in late December 2019 in China, and subsequent declaration by the WHO as a global pandemic in March 2020 [7] forced countries to implement strict hygiene regimes and social distancing measures. Extensive social distancing policies were put into place restricting people’s daily activities. While these restrictions helped slow the rate of infection, there may be concomitant negative effects as a result of limiting participation in normal daily activities such as walking and cycling for transport and leisure and access to many types of recreational activities such as team sports, gyms, fitness centers, and dancing classes.

Several studies have shown the impact of COVID-19 on healthy levels of PA, sedentary behaviour (SB) and sleep, collectively referred to as 24-h movement behaviours [8]. A recent systematic review which included 13 PA and 26 SB studies found that all studies reported decreases in PA and increases in SB, respectively, from before to during COVID-19 [9]. An Australian survey of 1500 participants found that nearly half reported a decrease in PA levels in April 2020 compared with before COVID-19 [10]. Negative changes in PA were associated with higher depression, anxiety and stress symptoms in this study. An international survey of PA including 1000 adults from Europe, Africa, Asia and the Americas showed that home confinement during the pandemic had a negative effect on all PA intensity levels [11]. Sitting (measured in hours/day) increased by 28.6%, Vigorous intensity PA decreased by 22.7%, and the number of days/week of walking decreased by 35% [11]. In Spain, participants reduced their weekly PA levels by 20% (~ 45.2 weekly minutes) during the first week of confinement compared with the previous week and the proportion who met the WHO guidelines decreased from 61 to 49% [12]. In Greece, a survey of 8495 adults found that time spent in daily occupational activity, active transport, and sporting activities reduced substantially, with overall PA decreasing by 16% [13]. In the United Kingdom, a survey involving 723 adults during a week of lockdown in April 2020 found that although 35% reported exercising less during COVID than before, 49% reported exercising more than before [14]. These results were similar when the study was extended to a larger sample (*N* = 2002) and prolonged until the 22nd of May with 40% reporting a decrease in PA, and 45% an increase in PA [15]. In an earlier global study, 19.1 million daily step count measurements were provided by 455,404 unique users from 187 countries, within 10 days of the pandemic declaration. There was a 5.5% decrease in mean steps (287 steps), and within 30 days, there was a 27.3% decrease in mean steps (1432 steps) [16].

Several studies have investigated the association between COVID-19 and changes in sleep habits, including sleep duration and perceptions of sleep quality. In the Spanish study described above the percentage of adults who slept for fewer than 6 h per day decreased [17]. In Jordan, it was reported that anxiety and depression during lockdown were associated with poorer sleep quality and shorter sleep duration [18]. A study of nearly 1000 participants in India found that compared to before lockdown, adults were going to bed and waking up later, sleeping less at night, and napping more during the day [19].

The Russian Federation has reported one of the largest number of COVID-19 infections (https://epidemic-stats.com/) [20]. Social distancing, travel bans, the cancellation of sporting and other mass participation events, and changes to work practices have dramatically affected daily life throughout the country. Major restrictions were introduced on the 28th March 2020 with the level of restrictions varied depending on the epidemiology of the virus from region to region. Cities with a large number of cases had the strictest restrictions, such as limitations on any outdoor activities. Further, citizens were required to possess an electronic pass to leave their house and this was only permitted for workers during work hours, and to access essential services such as medical or health care, to shop for groceries, or to visit parks and green zones (excluding outdoor activities entirely). In cities with a smaller number of cases, limitations were less strict especially with outdoor activities. Indoor sport activities were limited across the country and outdoor sports were restricted – depending on the situation in the region. Social distancing measures such as keeping a minimum 1.5 m between people were introduced, as well as a ban on any public gatherings of more than 50 people. On-line learning for schools and universities and recommendations for remote working for employees were introduced.

In the Russian Federation, population levels of PA are sub-optimal [21, 22]. This low prevalence of adequate PA is a main contributing factor to the high prevalence of overweight and obesity [23]. In addition, the prevalence of adequate sleep (defined by the US CDC and National Sleep Foundation as 7–9 h per night for adults) was only 37% [24]. However, the relationship between COVID-19 and perceived changes in PA and sleep is not known.

This study provides the first known data on the changes in PA and sleep habits during the period of COVID-19 among adults in the Russia Federation. Given the size of Russia, it was important to examine how these behaviours changed at a national level, given the variability in restrictions across regions.

The aim of this study was to examine the associated between COVID-19 and changes in levels of PA and sleep and to examine specific COVID-19 factors that may be related to changes in PA and sleep among adults in Russia.

The research questions were:

1. To what extent has PA and sleep changed as a result of the COVID-19 restrictions?
2. What COVID-19 factors were associated with these changes in PA and sleep?

## Methods

### Study design and population

The National Medical Research Center for Therapy and Preventive Medicine of the Ministry of Health of Russia (specifically the group of authors for this paper and an international co-author) conducted a national online-survey titled “Study of the impact of restrictions on PA of the population in self-isolation due to COVID-19.” An anonymous online survey was hosted on a Google online survey platform. A link to the electronic survey was distributed using social media sources (Facebook, Vkontakte and Odnoklassniki) and via institutional sources including email and public marketing. To increase regional participation a link to the electronic survey was send to the chief specialist in public health and preventive medicine in regional Ministries of Health who then distributed it at regional levels in Russia via a range of methods such as a shared link on the official web-pages and social media of regional Centers for public health and medical prevention and on regional Ministries of Health official web pages. The general public was asked to promote survey across their personal networks. The starting page of the survey informed participants about the aims and details of the survey. This method of data collection provides data from a convenience sample and whose population parameters cannot be controlled as is the case for probabilistic sampling. However, it was effective with respect to the research objectives because it allowed the broad dissemination of the survey during a period where, due to COVID-19, there were many restrictions on the collection of such data.

Eligible participants included all Russian adults aged 18 years and over. Data collection occurred between 26 April 2020 and 6 June 2020. This was the period of tightest restriction through the country, as from the 9th June 2020 there was a gradual releasing of restrictions depending on the regional context.

#### Survey development

The online survey was designed by a steering group at the National Medical Research Center for Therapy and Preventive Medicine of the Ministry of Health of Russia. Existing COVID-19 surveys from Canada, China, and the United Kingdom were used to inform development of the survey.

A 7-day self-report recall measure was selected as a method for PA assessment. Since the study was conducted during the challenging time of the pandemic-related lockdown, PA assessment was simplified to avoid the negative effect of the length of the questionnaire and its complexity on the response rate [25, 26].

The questionnaire contained 30 items and included mostly close-ended questions. Section 1 consisted of 10 general and context-related questions regarding demographic data and following self-isolation recommendations. Section 2 was designed to assess PA and sleep habits before COVID-19 and in the last 7 days during the pandemic. Section 3 evaluated how participants followed the COVID-19 preventive measures recommended by the Ministry of Health. Questions in Section 2 were presented in a differential format, to be answered directly in sequence regarding “before” and “during” confinement conditions. The full version of the questionnaire is available in the Supplementary file.

PA was assessed using eight items. Briefly, participants were asked to report, for before COVID and during the past 7 days, the number of days per week and the amount of time per day spent in vigorous-intensity activities, moderate-intensity activities, muscle-strengthening activities, calisthenic-type activities, and in walking. Total PA was calculated according to the WHO guidelines [4]: ≥150mins/week MPA or ≥ 75mins/week VPA or combination of MVPA, muscle strength activities ≥2 days/week. To calculate the proportion who met the first recommendation, we first calculated the number of minutes per week spent in vigorous-intensity activity, moderate-intensity PA and walking by multiplying the number of days spent in each of these activities by the time usually spent doing them. For the muscle strengthening, we added together the number of days of strength training and the number of days participating in basic calisthenic exercises (such as stretching, Zumba, yoga, pilates, Tai Chi) were undertaken.

For sleep habits and perceptions of sleep quality, participants reported, for before COVID and in the past 7 days, if they they felt they were getting enough sleep, if they had trouble falling asleep, and if they woke up earlier than they wanted.

### Data privacy and consent of participation

During the informed consent process, survey participants were assured all data would be used only for research purposes. Participants’ answers were anonymous and confidential according to Google’s privacy policy (https://policies.google.com/privacy?hl=en). Participants were not permitted to provide their names or contact information. Additionally, participants were able to stop participation and leave the questionnaire at any stage before the submission process; if they chose to do so their responses were not saved. Responses were saved only by clicking on the “submit” button at the end of the survey. By completing the survey, participants acknowledged their voluntary consent to participate in this anonymous study. This study was approved by the Ethics Committee of the National Medical Research Center for Therapy and Prevention of the Ministry of health of Russian Federation.

### Statistical analyses

#### Descriptive statistics

Descriptive statistics, including frequencies and percentages, were generated for categorical variables; means and standard deviations (SD) were generated for continuous variables. Data were analyzed in SPSS 20 (SPSS Inc., Chicago, IL, USA). Normality of the data distribution was examined using the Kolmogorov-Smirnov test.

#### Linear and logistic regressions

Linear regression was used to test the associations between changes in PA and selected COVID-19 factors. Crude estimates and estimates adjusted for gender were reported with 95% Confidence Intervals (CI). Logistic regression was used to test which factors had impact on meeting the PA recommendations (both muscles strengthening activities and minutes per week in MPA and VPA). Binary logistic regression analyses were conducted to investigate the association between categorical (dependent) and continuous or categorical (independent) variables. For these analyses ‘meeting PA Recommendation (150mins/week MPA or 75mins/week VPA)’ and ‘meeting muscle-strengthening activities recommendation (≥2 days/week)’ were specified as the dependent variables, and followed self-isolation recommendations, had access to outside/green zone, had an increase in the number of days per week with sleep problems, used digital or online PA resources, followed at least two relevant preventive measures from Ministry of Health, and geographic location were specified as independent variables. All variables in analysis for adjusted for age and sex. Statistical significance was set a priori at *P* < 0.05.

## Results

The characteristics of the study sample are presented in Table 1. A total of 2540 participants from 62 regions commenced the survey with 18 participants (0.7%) failing to complete and their data not being used in the analyses. Of the 2432 participants who completed the survey, 83% were females. Compared with males, females tended to be older, married, have children under 18 living with them, and in full-time employment, less likely to have completed higher education, and more likely to follow self-isolation recommendations. In terms of employment status, 1714 (70.5%) participants had a full-time job, 399 (16.4%) were students, 65 (2.7%) were unemployed, and 47 (1.9%) were retired.

**Table 1:** Sample characteristics

	Male (*n* = 328)	Female (*n* = 2104)	All (*n* = 2432)
Age (Mean, SD)	33.6 ± 14.9	38.2 ± 13.1	37.6 ± 13.4
Marital status, n (%)			
*Married*	145 (48.3)	1267 (64.2)	1412 (62.1)
*Single*	133 (44.3)	453 (23.0)	586 (25.8)
*Divorced*	19 (6.3)	178 (9)	197 (8.7)
*Widow/widower*	3 (1.0)	75 (3.8)	78 (3.4)
Live in urban area, n (%)	243 (74.1)	1482 (70.4)	1725 (70.9)
Have children under 18 living with them, n (%)	101 (30.8)	925 (44.0)	1026 (42.2)
Higher Education completion, n (%)	181 (55.2)	964 (45.8)	1145 (47.1)
Full-time employment status, n (%)	197 (60.1)	1517 (72.1)	1714 (70.5)
Followed self-isolation recommendation (completely or partially), n (%)	262 (79.9)	1898 (90.2)	2160 (88.8)
Current area of residence*,* n (%)			
*City*	243 (74.1)	1482 (70.4)	1725 (70.9)
*Village*	84 (25.6)	609 (28.9)	693 (28.5)
Type of residence, n (%)			
*Mansion, Townhouse*	85 (25.9)	635 (30.2)	720 (29.6)
*Flat, Hostel*	239 (72.9)	1457 (69.2)	1696 (69.7)
Own a pet dog, n (%)	73 (22.3)	570 (27.1)	643 (26.4)
Access to outdoors, n (%)	288 (87.8)	1932 (91.8)	2220 (91.3)
Access to a “green space”, n (%)	219 (66.8)	1486 (70.6)	1705 (70.1)
Use digital/online physical activity resources, n (%)	98 (29.9)	764 (36.3)	862 (33.6)
How COVID-19 affected your physical activity, n (%)			
*No effect*	107 (32.6)	717 (34.1)	824 (33.9)
*Fitness centre was closed*	97 (29.6)	380 (18.1)	477 (19.6)
*Could not leave house*	71 (21.6)	453 (21.5)	524 (21.5)
*I started participating in basic calisthenic exercises*^a^	49 (14.9)	418 (19.9)	467 (19.2)
*I started using a home exercise bike or treadmill*	30 (9.1)	163 (7.7)	193 (7.9)
*Other*	57 (17.4)	329 (15.6)	386 (15.9)
What COVID-19 measures were followed, n (%)			
*Wash hands more often*	286 (87.2)	1909 (90.7)	2195 (90.3)
*Avoid touching face*	209 (63.7)	1479 (70.3)	1688 (69.4)
*Avoid traveling*	211 (64.3)	1580 (75.1)	1791 (73.6)
*Maintain social distancing*	260 (79.3)	1679 (79.8)	1939 (79.7)
*Self-isolation*	262 (79.9)	1898 (90.2)	2160 (88.8)

^a^This was defined as exercises such as stretching, Zumba, yoga, Pilates, and Tai Chi

During the COVID-19 period, most participants completely or partially followed the self-isolation recommendations (*n* = 2160, 88.8%). The preventive measures followed most frequently were “wash hands more often” (90.3%) and “maintain social distancing” (79.7%). Over 90% of participants still had access to outdoor areas and 70% had access to a green space during the restriction period. Two-thirds of participants reported that COVID-19 affected their PA, mostly as a result of their fitness centre closing, of not being able to leave the house and of being able to undertake only simple calisthenic exercises at home. One-third of participants reported using online PA resources to help them be active, during this period.

Changes in sleep and PA from pre-COVID to during COVID are reported in Table 2. There was a significant decline in the number of days per week participants reported not getting enough sleep (3.21 ± 2.44 to 2.86 ± 2.57; *P* < 0.001) and participants also reported an increase in the number of days per week they had trouble falling asleep (1.70 ± 2.24 to 2.13 ± 2.48; *P* < 0.001). All PA outcomes declined significantly from pre- to during COVID. The average time spent in MPA and VPA each declined by around 12 min per day (42.43 ± 37.57 to 30.44 ± 35.35 and 37.79 ± 37.80 to 26.56 ± 34.69, respectively [all *P* < 0.001]). The number of minutes per day spent walking decreased by around 20 min from 60.5 ± 38.66 to 40.83 ± 38.6 (*P* < 0.001).

**Table 2:** Changes in sleep and physical activity from pre- to during COVID-19

	Males			Females			Total		
	Pre-COVID	During COVID	*P*-value	Pre-COVID	During COVID	*P*-value	Pre-COVID	During COVID	*P*-value
Number of days per week not getting enough sleep (M, SD)	2.88 ± 2.39	2.66 ± 2.56	< 0.001	3.26 ± 2.45	2.9 ± 2.57	< 0.001	3.21 ± 2.44	2.86 ± 2.57	< 0.001
Number of days per week having trouble falling asleep sleep (M, SD)	1.74 ± 2.32	2.14 ± 2.54	< 0.001	1.7 ± 2.23	2.13 ± 2.47	< 0.001	1.7 ± 2.24	2.13 ± 2.48	< 0.001
Number of days per week waking up earlier than wanted (M, SD)	2.65 ± 2.64	2.45 ± 2.61	< 0.001	2.62 ± 2.62	2.62 ± 2.6	0.905	2.6 ± 2.62	2.59 ± 2.6	0.505
Days per week engaged in MPA	3.4 ± 2.39	2.47 ± 2.38	< 0.001	2.92 ± 2.44	2.1 ± 2.32	< 0.001	2.99 ± 2.44	2.15 ± 2.33	< 0.001
Average time per day spent in MPA, mins	52.5 ± 38.5	37.59 ± 37.59	< 0.001	40.87 ± 37.18	29.32 ± 34.87	< 0.001	42.43 ± 37.57	30.44 ± 35.35	< 0.001
Days per week engaged in VPA	2.6 ± 2.29	2.03 ± 2.25	< 0.001	2.18 ± 2.19	1.74 ± 2.18	< 0.001	2.24 ± 2.21	1.78 ± 2.19	< 0.001
Average time per day spent in VPA, mins	48.98 ± 40.81	32.88 ± 37.53	< 0.001	36.05 ± 37.01	25.57 ± 34.13	< 0.001	37.79 ± 37.80	26.56 ± 34.69	< 0.001
Days per week spending walking (M, SD)	5.22 ± 2.25	3.58 ± 2.67	< 0.001	5.37 ± 2.09	3.78 ± 2.64	< 0.001	5.35 ± 2.12	3.76 ± 2.64	< 0.001
Average time per day spent walking (M, SD), mins	63.48 ± 38.66	40.75 ± 38.05	< 0.001	60.04 ± 38.65	40.84 ± 38.7	< 0.001	60.5 ± 38.66	40.83 ± 38.6	< 0.001
Number of days per week doing resistance training	1.86 ± 2.09	1.52 ± 2.1	< 0.001	1.12 ± 1.79	0.96 ± 1.82	< 0.001	1.22 ± 1.85	1.04 ± 1.87	< 0.001
Number of days per week spent doing exercises such as gymnastics, yoga	1.27 ± 2.12	1.1 ± 1.98	< 0.001	1.55 ± 2.11	1.45 ± 2.17	0,002	1.51 ± 2.11	1.41 ± 2.15	0.001
Meeting PA Guidelines, %									
*≥150mins/week MPA or*	45.7%	31.1%	< 0.001	35.0%	20.8%	< 0.001	36.4%	22.2%	< 0.001
*≥75mins/week VPA or*	56.7%	39.9%	< 0.001	45.5%	29.7%	< 0.001	47.0%	31.0%	< 0.001
*Combination of MVPA*	78.0%	57.9%	< 0.001	66.0%	47.3%	< 0.001	67.6%	48.8%	< 0.001
*Muscle strength. Activities ≥ 2 days/week*	57.9%	47.3%	< 0.001	52.2%	44.2%	< 0.001	53.0%	44.6%	< 0.001

*PA* Physical activity, *MPA* Moderate-intensity physical activity, *VPA* Vigorous- intensity physical activity, *MVPA* Moderate- and vigorous intensity physical activity

The proportion of participants who met the WHO Guidelines for any type of PA declined from 68 to 49% (*P* < 0.001). The proportion who participated in muscle strengthening activities for 2 or more days per week declined from 53 to 45% (*P* < 0.001).

Associations between changes in days and time spent in PA and sleep habits and selected COVID-19 factors are reported in Table 3. Factors consistently associated with a greater decline in minutes per week spent in VPA, in MPA and in walking included an increase in number of days with sleep problems (β = − 28, 95%CI − 41 to − 15; β = − 42, 95%CI − 59 to − 29; and β = − 83, 95%CI − 104 to − 62, respectively), closure of fitness centre/gym (β = − 99, 95%CI − 116 to − 84; β = − 77, 95%CI − 96 to − 59; and β = − 41, 95%CI − 66 to − 15, respectively) and not being able to leave the house for PA (β = − 93, 95%CI − 109 to − 78; β = − 123, 95%CI − 141 to − 105, and β = − 217, 95%CI − 243 to − 192, respectively). Factors associated with a smaller decline in minutes per week in VPA and MPA included using digital or online resources (β = 19, 95%CI 5 to 33 and β = 23, 95%CI 7 to 38, respectively) and having access to a home gym (β = 38, 95%CI 15 to 62 and (β = 33, 95%CI 7 to 60, respectively). Factors associated with a greater decline in days per week participating in muscle strengthening activities included closure of fitness centre/gym (β = − 99, 95%CI − 116 to − 84) and not being able to leave the house for PA (β = − 93, 95%CI − 109 to − 78). Factors associated with a smaller decline in days per week participating in muscle strengthening activities included using digital or online resources (β = 0.4, 95%CI 0.2 to 0.6), being able to participate in simple calisthenics at home (β = 1, 95%CI 0.8 to 1.3), and having access to a home gym (β = 0.9, 95%CI 0.5 to 1.2). Following self-isolation recommendations (β = − 0.6, 95%CI − 0.8 to − 0.3) and having access to a home gym (β = − 0.1, 95%CI − 0.4 to 0.2) were associated with a greater reduction in the number of days per week participants reported not getting enough sleep. In contrast, having children under 18 years of age in the residence (β = 0.4, 95%CI 0.2 to 0.6) and not being able to leave the house for PA (β = 0.2, 95%CI 0.02 to 0.4) were associated with a smaller reduction in the number of days per week participants reported not getting enough sleep.

**Table 3:** Associations between changes in time spent in physical activity and sleep and selected COVID-19 factors

	Change (In the last 7 days minus Before COVID-19)									
	Mins per week VPA		Mins per week MPA		Mins per week walking		Days per week muscle strengthening activities		Days per week not getting enough sleep	
	β	95% CI	β	95% CI	β	95% CI	β	95% CI	β	95% CI
Had children U18 living with you	−0.64	−1.28, 0	−0.74	−1.48, 0	− 0.2	− 1.22, 0.82	− 0.01	− 0.02, 0.01	0.414	0.25, 0.58
Followed self-isolation recommendation	4.02	−15.96, 23.99	− 13.94	−36.99, 9.11	−84.01	− 115.73, −52.29	0.09	− 0.22, 0.4	− 0.555	− 0.82, − 0.29
Owned a pet dog	− 0.36	−1.56, 0.85	− 0.83	−2.22, 0.56	− 0.9	−2.81, 1.02	−0.01	− 0.03, 0.01	−0.002	− 0.21, 0.20
Had access to the outside	0.99	−0.01, 2.00	0.64	−0.53, 1.8	0.59	−1.01, 2.19	−0.001	−0.02, 0.01	− 0.151	−0.44, 0.14
Had access to a “green space”	−0.38	−1.10, 0.34	−0.33	− 1.17, 0.5	− 0.6	− 1.74, 0.54	0.004	−0.01, 0.015	− 0.098	−0.29,0.09
Had increase in number of days per week with sleep problems	−28.10	−41.09, − 15.11	−44.32	−59.31, 29.34	−82.90	− 103.53, −62.27	−0.03	− 0.23, 0.17	1.302	1.13,1.47
Used digital or online PA resources	18.85	5.16, 32.55	22.62	6.81, 38.42	8.28	−13.48, 30.03	0.42	0.21, 0.63	−0.44	−0.62, − 0.26
Followed at least two relevant preventive measures from Ministry of Health	12.79	−17.99, 43.57	6.80	−28.72, 42.32	−28.21	−77.10, 20.67	0.29	−0.19, 0.76	−0.091	− 0.49,0.31
Fitness centre/gym closed	−99.56	−115.61, −83.51	− 77.03	−95.55, −58.50	−40.74	−66.23, − 15.24	−1.36	− 1.61,-1.11	0.044	−0.16,0.25
Couldn’t leave the house for PA	−93.41	−109.21, − 77.62	− 122.75	−140.98,-104.53	−217.48	− 242.56, − 192.39	−1.25	− 1.49, − 1.01	0.231	0.02,0.44
Able to participate in calisthenic activities at home	19.42	2.80, 36.05	1.30	−17.89, 20.48	−21.92	−48.33, 4.48	1.07	0.82, 1.33	−0.473	− 0.69, − 0.256
Had a home gym	38.46	15.33, 61.59	33.24	6.54, 59.93	−20.68	−57.42, 16.05	0.88	0.53, 1.24	−0.134	−0.43,0.17
Live in a Metropolis/city	−16.46	−43.23, 10.30	−8.36	−39.25, 22.52	−5.12	−47.63, 37.38	−0.13	−0.54, 0.28	− 0.081	−0.43,0.27
Live in a flat/apartment	−11.70	−27.92, 4.51	−25.95	−44.67, −7.24	−38.44	−64.19, − 12.68	0,01	− 0.24, 0.26	− 0.123	−0.34,0.1
Live in urban area (population)	15.18	− 12.38, 42.74	3.33	−28.49, 35.13	− 12.29	− 56.06, 31.48	0.07	−0.36, 0.49	−0.337	− 0.70,0.02

All analyses adjusted for sex and age

*PA* Physical activity, *MPA* Moderate-intensity physical activity, *VPA* Vigorous-intensity physical activity

Associations between meeting WHO Global PA and muscle-strengthening recommendations and selected COVID-19 factors are reported in Table 4. Compared with those who did not use online PA resources, those who did were 1.4 (95%CI 1.3, 1.5) and 1.9 (95%CI 1.8, 2.1) times more likely to meet the recommendations for PA and for muscle-strengthening activities, respectively. Compared with those who did not have access to a green space, those who did were more likely to meet the PA (OR = 1.2, 95%CI 1.1, 1.2) and muscle strengthening (OR = 1.1, 95%CI 1.1, 1.2) recommendations. Those who owned a pet dog (OR = 1.2, 95%CI 1.1, 1.3) and those who followed the self-isolation rules (OR = 1.3, 95%CI 1.2, 1.4) were more likely to meet the PA and muscle strengthening recommendations, respectively, than those who did not. Conversely, compared with their urban counterparts, rural adults were less likely to meet the PA recommendation (OR = 0.9, 95%CI 0.8, 0.9).

**Table 4:** Associations between meeting WHO Global PA Recommendations and selected COVID-19 factors

	Meeting PA Recommendation (150mins/week MPA or 75mins/week VPA)		Meeting muscle-strengthening activities recommendation (≥2 days/week)	
	%	OR (95%CI)	%	OR (95%CI)
Follow self-isolation recommendation				
No (ref)	88.0%	0.95 (0.85–1.05)	92.2%	1.28 (1.17–1.4)
Yes				
Own a pet dog				
No (ref)	32.5%	1.23 (1.13–1.33)	27.3%	1.04(0.95–1.12)
Yes				
Had access to outside				
No (ref)	92.8%	1.12 (1.01–1.24)	91.2%	0.99 (0.87–1.12)
Yes				
Had access to a green space				
No (ref)	75.4%	1.17 (1.09–1.24)	73.7%	1.14 (1.06–1.23)
Yes				
Had increase in number of days per week with sleep problems				
No (ref)	66.1%	0.88 (0.83–0.94)	62.9%	0.95 (0.88–1.02)
Yes				
Used digital or online PA resources				
No (ref)	47.0%	1.40 (1.3–1.51)	52.0%	1.93 (1.75–2.13)
Yes				
Following at least two relevant preventive measures from Ministry of Health				
No (ref)	4.2%	1.00 (0.86–1.17)	4.6%	1.08 (0.89–1.30)
Yes				
Geographic location				
Urban (ref)	25.7%	0.89 (0.82–0.96)	23.6%	0.95 (0.87–1.04)
Rural				
Live in a flat/hostel				
No (ref)	61.1%	0.8 (0.74–0.87)	65.5%	0.91 (0.84–0.98)
Yes				

All analyses adjusted for sex and age

*PA* Physical activity, *MPA* Moderate-intensity physical activity, *VPA* Vigorous- intensity physical activity

## Discussion

We found that as a result of COVID-19 restrictions in Russia, there were perceptions among adults that their participation in PA and their sleep duration had significantly declined compared with pre-COVID levels. Not being allowed to leave the house for PA and the closure of fitness centres were policies associated with greater declines in PA and sleep. Conversely, those individuals who could access a green space or who participated in activities at home – using online resources or with the necessary equipment – showed much smaller declines in PA and sleep. In addition, we found that those who lived in apartments and in rural areas were more likely to be adversely affected in terms of their participation in PA.

In our study the number of days per week that participants got enough sleep decreased while the number of days per week that participants had trouble falling asleep increased. These results are consistent with studies in China [27], and Italy [28, 29] showing the negative impact of COVID-19 home confinement on sleep, but in contrast with Spanish survey where overall duration of sleep increased without worsening its quality [17]. In Russia, COVID-19 upended daily routines in a number of ways: more people were working from home, meals times were altered, SB – especially screen time – increased. These factors, in addition to the social distancing requirements, likely resulted in a disruption to circadian rhythms [30]. Home confinement is associated with reduced levels of PA which, in addition to the social isolation, may increase stress levels and disrupt night-time sleep. Physiological factors such as reduced sunlight exposure and weaker light–dark cycles as a result of less time spent outdoors may also have affected sleep and circadian rhythms [31].

We found that the restrictions during COVID may have been associated with a reduction in number of days per week and number of hours per day spent in PA and a reduction in all types of activities. It is important to note that the number of days participating in resistance or strength training also decreased significantly as did the proportion who met the 2020 WHO recommendation to do muscle-strengthening activities on at last 2 days per week. During home confinement, such activities can be performed using digital or online resources, which we found was positively associated with a higher number of days per week of muscle strengthening activities. Despite an increased offering of digital or online resources that could be accessed at home, participants perceived that they were not able to maintain their normal pre-COVID PA levels. Those who did take up the offering and use such resources were more likely to meet PA recommendations. More support needs to be provided to those who were not able to access these resources, which may have been exacerbated by the higher increased levels of stress and uncertainty.

Providing opportunities for PA is important in building strong immune systems and reducing the susceptibility to infection. A recent study using Mendelian randomization demonstrated that higher levels of PA was associated with a lower probability of being admitted as an outpatient for COVID-19 [32]. Conversely, a decline in PA in patients with a chronic noncommunicable disease increase both the risk of COVID-19 as well as the risk of cardiovascular and other adverse events [33].

However, the extent to which changes in PA participation may be associated with the COVID-19 pandemic is dependent upon the confinement policies of individual governments. For example, in China different policies at regional levels was associated with differences in PA participation [34].

We found that a perceived increase in sleep problems was associated with a perceived greater decline in PA and less likelihood to meet the PA guidelines. This reinforces how PA and sleep are interrelated, which is consistent with evidence from systematic reviews demonstrating the association between sleep and exercise. Exercise promotes increased sleep efficiency and duration regardless of the mode and intensity of activity, especially in populations suffering from disease [35].

We identified several factors that were associated with healthier levels of PA and sleep habits during COVID-19. These suggest that the impact of the pandemic has not been uniform among Russian adults. People living in urban area were less likely to achieve the PA recommendations, which is consistent with findings from before the pandemic and demonstrates that those living in urban areas are more active that their rural counterparts [21]. Living in a detached house, owning a dog, having a home gym were all favorably associated with healthy movement behaviours. In addition, having access to a green space was positively associated with PA. This information may be helpful to policymakers of population sub-groups who are at highest risk of being inactive during COVID in potentially informing what should be considered when planning a response to provide opportunities to be active while at the same time adhering to social distancing requirements. Participants living in a house versus an apartment may have easier access to front or back yards for outdoor play and PA [36]. Families who had a dog had higher PA and outdoor time. A recent systematic review also showed that dog-related interventions increased PA [37].

To the best of our knowledge, this is the first published study to report on PA and sleep among Russian adults during the COVID-19 pandemic. Another international online survey on PA had similar findings but did not include participants from the Russian Federation [11]. Other studies from Australia [10], Poland [38], Spain [12], Greece [13] reported similar reductions in PA and increases in SB during lockdown in adults. In United Kingdom survey 35% participants reported less than before exercising, but 49% more than before [14].

There is a need to address the impact of COVID-19 on healthy levels of movement behaviours and subsequent NCD risk, including using modern technologies (on-line) as part of a suite of strategies [39]. To prevent the unintended consequences of COVID-19 restrictions and ‘stay home’ advice on PA and sleep – and as a corollary mental and social health, a balance is needed between preventing the spread of infection and providing opportunities for people to participate in healthy levels of movement behaviours.

Our results can be used to further research and development in public health promotion in Russia during the COVID-19 pandemic. Health promotion campaigns aimed at informing the population about the risks of physical inactivity are recommended. Evidence also suggests that web -[39] and app-based [40] interventions that people can access in their home might be especially beneficial if participants are motivated to adhere to the requirements. Some technology and social media have used gamification to overcome challenges in adherence, fitness influencers on Instagram can be one of the drivers of increasing PA [41]. Further enhancements such as providing opportunities for social interaction should also be considered.

## Limitations

While there are a number of strengths of the present study, such as the large sample size, and the timing of data collection relative to lockdown restrictions in Russia, there are a number of limitations. First, our study was cross-sectional meaning participants perceived the changes in their PA and sleep from before to during COVID-19. As such, participants may have been more likely to overstate the changes in PA and sleep in the absence of any true baseline data. Second, all data were self-reported and subject to recall bias such as overestimation of time spent in PA and in sleep. Although the sleep questions were modified from questions found in other questionnaires [42–44], these items were not able to be validated in a Russian population before the survey was administered during the initial stage of COVID-19. They also only ask about one aspect of sleep quality and insomnia, namely difficulty in falling asleep and in getting enough sleep. Other aspects related to insomnia and sleep quality would provide a more detailed description of the impact of COVID-19 on these important aspects of sleep. Third, our sample included an under-representation of males, which although unfortunate is consistent with other COVID-19 survey research among adults [10, 13, 14, 38]. As such, these data may not be reflective of males living in Russia. In our opinion this survey can be generalized to some groups in the Russian population, predominantly women of young and middle age from five regions who actively use Internet (Tver, Tatarstan, Irkutsk, Sakhalin, and Bashkortostan). This group could be a target for any or web- or app-based interventions to promote healthy levels of PA and sleep considering that it will likely impact not only on them but potentially to members of their families, children and male partners. Fourth, we adjusted for sex and age but not for other covariates such as socioeconomic status in the analyses. Finally, during the COVID-19 outbreak, the main instruments used to investigate perceived changes in PA and sleep habits were on-line surveys [11, 45]. This method has limitations but was the preferred method available during this period, being easier to collect data compared with a telephone survey. Online research is therefore a recommended approach if the aim is to reach a large group of participants in a short period of time, ensuring their safety under pandemic conditions [46].

## Conclusion

Results of on-line cross-sectional surveys can be important to guide the development of interventions aimed to improve negative lifestyle behaviours associated with COVID-19 confinement. Health promotion strategies directed at promoting positive health-related behaviors should be introduced to counter the negative impact of the pandemic. Ongoing evaluation of the impact of different levels of restrictions – which will be present in some jurisdictions for an extended period of time – on health behaviors is necessary to inform these targeted health promotion strategies. Healthy levels of PA and sleep also affect the immune system through promoting healthy circadian rhythms and as such might serve as a protective strategy against infectious diseases.

This study has implications for policymakers in Russia. It demonstrates the need for efforts to stimulate wider use of on-line resources for PA, and to consider the possibility of re-opening gyms and fitness centres, with all possible safety contingencies, during the period of restrictions. Local councils should consider the importance of providing access to green spaces as part of their COVID-19 policies, especially if accompanied by infection control measures such as social distancing and wearing face masks. Special considerations should also be made for high risk groups such as older adults and people living with chronic diseases.

Future studies should also evaluate the longer-term associations between the COVID-19 virus outbreak and recovery on PA, sedentary and sleep behaviours. To develop targeted health promotion strategies in Russia, it would be useful to identify province-specific or geographic differences influencing health behaviours.

## Supplementary Information

**Additional file 1.** Questionnaire.

## Data Availability

All data generated or analysed during this study are included in this published article [and its supplementary information files].

## Abbreviations

COVID-19: Coronavirus Disease 2019
WHO: World Health Organization
PA: Physical activity
MPA: Moderate physical activity
VPA: Vigorous-intensity physical activity
SB: Sedentary behaviour
