# Supplementary material for: Changes in physical activity and sleep habits among adults in Russian Federation during COVID-19: a cross-sectional study
Source: BMC Public Health. 2021 May 11;21:893. doi: 10.1186/s12889-021-10946-y (PMC8111050; doi:10.1186/s12889-021-10946-y)
Supplement: Supplementary file 1 — Additional file 1. Questionnaire. [file 12889_2021_10946_MOESM1_ESM.docx]

Supplement file

Changes in physical activity and sleep among adults in Russian Federation during COVID-19: a cross-sectional study

Anna V. Kontsevaya¹, Dinara K. Mukaneeva¹, Azaliia O. Myrzamatova¹, Anthony D. Okely², Oxana M. Drapkina¹

¹Department of Public Health Promotion, National Medical Research Center for Therapy and Preventive Medicine of the Ministry of Health of Russia, Moscow, Russia

²Early Start and Illawarra Health & Medical Research Institute, University of Wollongong, Wollongong, NSW, Australia

Correspondence to Anna V. Kontsevaya, MD PhD, Deputy director on science and analytics, National Medical Research Center for Therapy and Preventive Medicine of the Ministry of Health of Russia, bld. 10, Petroverigskiy lane, Moscow, Russia, 101990. Email: koncanna@yandex.ru

+ 7 915 197 87 07

Final Questionnaire

| **Place of residence and date** | | **Answer** |
| --- | --- | --- |
| 1 | **What is your region?** |  |
| 2 | **City** |  |
| 3 | **Date of filling out the questionnaire** | └─┴─┘ └─┴─┘ └─┴─┴─┴─┘ DD. MM. YYYY. |

| **Socio - demographic characteristic** | | **Answer** | | |
| --- | --- | --- | --- | --- |
| 4 | **Sex** | Male  Female  Prefer not to answer | |  |
| 5 | **Age** | └─┴─┘ | | |
| 6 | **Marital status** | Married  Single  Divorced  Widow/widower  Prefer not to answer | |  |
| 7 | **Do you have children under the age of 18 living with you?** | Yes  No  Prefer not to answer | |  |
| 8 | **What education do you have?** | Senior High School  Сollege  Higher education  Master's degree  Prefer not to answer | |  |
| 9 | **What is your current employment status?** | Student  Full-time employment  Part-time employment  Self employed  Retired  On disability  Unemployed  Prefer not to answer | |  |
| 10 | **Do you follow self-isolation recommendation?** | Yes completely  Yes, partially  Leading the same way of life, as before the distribution of Covid-19  I prefer not to answer | |  |
| **Habitat** | | **Answer** | | |
| 11 | **You currently live in ...** | Metropolis  City  Suburb  Countryside  Remote or Northern areas  Prefer not to answer | |  |
| 12 | **You live in…** | Mansion  Townhouse  Flat  Hostel  Other  Prefer not to answer | |  |
| 13 | **Do you have a pet dog?** | Yes  No  Prefer not to answer | |  |
| 14 | **Do you have access to the outside (for example: a balcony, porch, courtyard) in your home?** | Yes  No  Prefer not to answer | |  |
| 15 | **Do you have access to the “green zone” (for example: backyard, courtyard, communal garden) in your home?** | Yes  No  Prefer not to answer | |  |
| 16 | **How far is the nearest green area Outside of your home? (e.g. park, dog walk, walking trail, garden, woodlands, nature reserve, etc.)** | └─┴─┘ ml. | | |
| **Physical activity and sleep** | | **Answer** | | |
| 17 | **How many days a week did you feel you were not getting enough rest or sleep?** | Before Covid-19: number of days └─┘  In the last 7 days: number of days └─┘ | | |
| 18 | **How many days a week did you have trouble falling asleep?** | Before Covid-19: number of days └─┘  In the last 7 days: number of days └─┘ | | |
| 19 | **How many days a week did you wake up earlier than you wanted?** | Before Covid-19: number of days └─┘  In the last 7 days: number of days └─┘ | | |
| 20 | **How many days a week did you engage in *high-intensity physical activity*, such as digging, aerobics, or fast Cycling, that significantly increases your breathing or heart rate?** | Before Covid-19: number of days └─┘    In the last 7 days: number of days └─┘ | | |
| 21 | **How much time did one of these days engage in *high-intensity physical activity*?** (indicate hours and minutes) | **Before Covid-19:**  Not involved  Less than 30 min 30-60 min 61-120 min More than 120  **In the last 7 days:**  Not involved  Less than 30 min 30-60 min 61-120 min More than 120 | | |
| 22 | **How many days a week did you engage in *moderate physical activity*, for example, running, cycling?** | Before Covid-19: number of days └─┘    In the last 7 days: number of days └─┘ | | |
| 23 | **How much time did you usually spend on one of these days doing *moderate physical activity***? (indicate hours and minutes) | **Before Covid-19:**  Not involved  Less than 30 min 30-60 min 61-120 min More than 120  **In the last 7 days:**  Not involved  Less than 30 min 30-60 min 61-120 min More than 120 | | |
| 24 | **How many days a week did you do *strength training*, such as weight lifting, aerobics or weightlifting?** | Before Covid-19: number of days └─┘    In the last 7 days: number of days └─┘ | | |
| 25 | **How many days a week did you perform exercises such as gymnastics, yoga, balance exercise?** | Before Covid-19: number of days └─┘    In the last 7 days: number of days └─┘ | | |
| 26 | **How many days a week did you *walk* more than 10 minutes at a time?** | Before Covid-19: number of days └─┘    In the last 7 days: number of days └─┘ | | |
| 27 | **How much time do you usually spend *walking* on a normal day?** (indicate hours and minutes) | **Before Covid-19:**  Not involved  Less than 30 min 30-60 min 61-120 min More than 120    **In the last 7 days:**  Not involved  Less than 30 min 30-60 min 61-120 min More than 120 | | |
| 28 | **Did the onset of the COVID-19 pandemic affect your physical activity?** (select all that apply, in your opinion) | No effect  My fitness center / gym closed  I can’t leave the house for sports  I became a simple gymnastics  I have a home gym  Other  I prefer not to read |  | |
| 29 | **Do you use digital or online physical activity resources? For example, a YouTube exercise video or fitness app?** | Yes  No  Prefer not to answer |  | |
| **Covid-19 Prevention** | | **Answer** | | |
| 30 | **What** **Covid-19 preventive measures recommended by the Ministry of Health do you personally follow at this time?** (select all that apply, in your opinion) | Wash hands more often  Avoid touching the face.  Avoid traveling  Keep social distance  Self- isolation  Other  I prefer not to answer | |  |
